# Social Influence as a Driver of Engagement in a Web-Based Health Intervention

**DOI:** 10.2196/jmir.1957

**Published:** 2012-02-22

**Authors:** Josée Poirier, Nathan K Cobb

**Affiliations:** ^1^MeYou Health, LLCBoston, MAUnited States; ^2^Division of Pulmonary & Critical CareDepartment of MedicineGeorgetown University Medical CenterWashington, DCUnited States; ^3^Department of OncologyGeorgetown University Medical Center/Lombardi Comprehensive Cancer CenterWashington, DCUnited States; ^4^Department of Health, Behavior and SocietyJohns Hopkins Bloomberg School of Public HealthBaltimore, MDUnited States

**Keywords:** Web-based health interventions, engagement, social networks, social influence

## Abstract

**Background:**

Web-based health interventions can drive behavior change, but their effectiveness depends on participants’ usage. A well-recognized challenge with these interventions is nonusage attrition or weak engagement that results in participants receiving low doses of the intervention, negatively affecting outcomes. We present an approach based on the theoretical concepts of social influence and complex contagion in an effort to address the engagement problem in a specific, commercial, online behavior change intervention.

**Objective:**

To examine the relation between social ties and engagement within a specific online intervention. The aims were (1) to determine whether experiencing the intervention socially influences engagement, such that individuals with social ties show higher engagement than those without ties, and (2) to evaluate whether complex contagion increases engagement—that is, whether engagement increases as the number of ties an individual has in the intervention increases.

**Methods:**

We analyzed observational data from 84,828 subscribed members of a specific Web-based intervention, Daily Challenge. We compiled three measures of engagement for every member: email opens, site visits, and challenge completions (response to action prompts). We compared members with and without social ties within the intervention on each measure separately using 2-tailed independent-sample *t* tests. Finally, we performed linear regressions with each simple engagement measure as the dependent variable and number of social ties as the independent variable.

**Results:**

Compared with those without social ties, participants with social ties opened more emails (33.0% vs 27.2%, *P* < .001), visited the website more often (12.6 vs 6.7 visits, *P* < .001), and reported completing more of the actions they were prompted to perform (11.0 vs 6.1 actions, *P* < .001). Social ties were significant predictors of email opens (beta = 0.68, *P* < .001), site visits (beta = 1.52, *P* < .001), and reported action completions (beta = 1.32, *P* < .001).

**Conclusions:**

Our initial findings are higher engagement in participants with social ties in the program and are consistent with the view that social influence can drive engagement in a Web-based health intervention.

## Introduction

Although Web-based health interventions can drive behavior change across multiple conditions [1], their effectiveness highly depends on participants’ usage. Adherence remains a well-recognized challenge with Web-based interventions, with rapidly decaying amount of exposure to program content (nonusage attrition; [2]) presenting a significant barrier to the development of effective systems. For instance, many Web-based interventions reported in the literature are affected by rapid attrition [2-4] and suboptimal consumption of program content, as measured by site visits and time spent on the site [5-7]. Despite this, such systems have also demonstrated a dose response, with increasing levels of adherence associated with improved outcomes [8-11]. Capitalizing on the potential of these systems to reach large proportions of the population will require solving the twin problems of adherence and ongoing consumer engagement.

Social influence may offer a solution to problematically weak engagement and adherence to an intervention as designed. The term refers to the ability of connected individuals to affect one another’s thoughts, ideas, and behaviors. Social influence contributes to the spread of behaviors through social contact [12,13], such that individuals adopt a new behavior more readily if their social ties display it. This effect may reflect individuals’ reliance on the actions of others to determine the appropriate behavior in a given situation (informational social influence), or their underlying desire to conform to the expectations of others (normative social influence) [14]. Thus, in a health intervention, facilitating interactions between participants and exposing them to one another’s activity may induce high behavior-adoption rates. When applied to program usage behavior, this approach could harness social influence to improve engagement in Web-based interventions and ultimately affect behavioral change outcomes. The social approach has been successful in previous Web-based health interventions (eg, QuitNet; [15]) and may be the most promising in terms of cost effectiveness and success rate [16].

In addition to their potential scalability at a minimal cost, social Web-based interventions can magnify the potential effect of social influence by markedly increasing social contact. The phenomenon of complex contagion has been reported across various domains such as technology, fashion, migration, urban legends, and, more recently, health [17,18]. It refers to the observation that individuals who receive social reinforcement from multiple contacts tend to adopt behaviors in higher numbers and do so more rapidly [17]. In social Web-based interventions, social reinforcement can occur particularly frequently. The multiplicity of ties a participant has increases the probability that he or she will be repeatedly exposed to others engaging in a given behavior. Hence, the very nature of social interventions offers a favorable environment for complex contagion to facilitate the adoption of a desired behavior such as high program usage.

In this study, we describe one real-world implementation in which theoretical concepts of social influence and complex contagion are applied to address the engagement problem. We present evaluation results of this approach, used to drive program usage in a specific, commercial, online health and wellness intervention.

This intervention, named Daily Challenge and delivered primarily on the Web and by email, allows members to form connections with other members, effectively building an individual social network within the intervention. The network provides various forms of social influence that may increase engagement: social proof, accountability, and support. The members’ activity is visible to their social network, and vice versa. Therefore, one can observe a contact (a connection or friend in the system) displaying a behavior (such as returning to the site, forming a new friendship, trying a new feature, or reporting having completed a challenge) within the intervention and adopt it as well (social proof). Further, the transparency of activity over every tie renders individuals accountable to every person in their network, potentially altering behavior. The visibility of members’ activity in the intervention enables their ties to detect and act on others’ struggles or inactivity by providing support and direct, personalized nudges (formalized communications, similar to a “wink” or “poke” in other products). Similarly, individuals can offer positive support to their connections in the form of encouragement, companionship, and information. We hypothesized that this combination of social proof, social accountability, and social support would increase engagement in the program.

The goal of this study was to explore the relation between social ties and engagement within this specific online intervention. Our aims were 2-fold: (1) to determine whether experiencing the intervention socially influences engagement, such that individuals with social ties show higher engagement than those without ties, and (2) to evaluate whether complex contagion increases engagement—that is, whether engagement increases as the number of ties an individual has in the intervention increases.

## Methods

### Intervention Description

**Figure 1:**
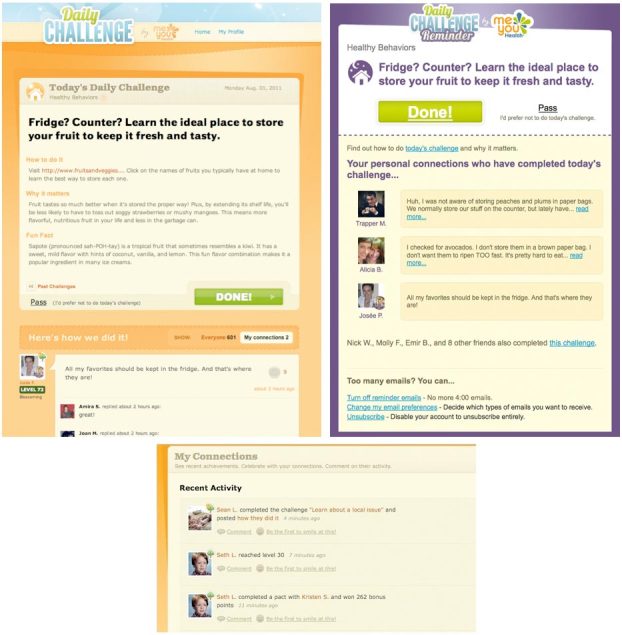
Screenshots of the Web-based intervention Daily Challenge. Top left: A challenge with its Done button, as seen on the site. Top right: An email reminder with social proof. Bottom: Activity stream in which members can follow their connections’ activity, including challenge completions.

Daily Challenge [19] is a publicly available health and wellness intervention designed to assist individuals in making small, health-related, positive changes to improve their overall well-being. The intervention, which undergoes continual development, is based on work demonstrating that the adoption of small behavioral changes across the larger population can have a significant public health benefit [20,21]. Individuals can register through Facebook. Each day after registration they receive, most commonly via email, a suggestion of a small action they can realistically accomplish that day. For example, an individual might be challenged to take the stairs at work, or to review the salt content in today’s lunch. Information on how to complete the day’s action and how it relates to well-being accompanies the suggestion, which is presented as a challenge for the day. Members can report that they have completed the challenge by clicking a Done button via a link in the email or on the website directly (Figure 1, top left). Members who do not acknowledge completion by 4 PM local time receive an optional reminder (Figure 1, top right).

Once they indicate they have completed the challenge, members are prompted to share how they did it (Figure 1, top left), either with everyone in the local (Daily Challenge) community, or solely with their Daily Challenge connections. These social connections are formed within Daily Challenge. To establish a connection, a member sends a friendship request to the member with whom he or she would like to connect. The other member may be an extant social tie (eg, a family member, friend, colleague, or Facebook friend) or someone the member met through Daily Challenge and might have interacted with in the public threads. If the request is accepted, the connection is formed. All connections require confirmation from the other party and are thus reciprocal.

Members with connections have access to the primary social features in the program: they can view the private and public “how I did it” notes of their personal connections (Figure 1, top left); they can send encouragement to a personal connection; or they can enter a pact to complete challenges together for 5 consecutive days. Additionally, connected members can follow each others’ activity more closely. Members can easily see when their connections complete challenges—among other actions—in an activity stream (Figure 1, bottom). The reminder emails also detail which of the members’ ties have completed the day’s challenge and how they did it (Figure 1, top right). Thus, members do not need to return to the site to be exposed to social proof.

As Daily Challenge is an operating, real-world intervention, maximizing participation rates is paramount. As such, minimal demographics beyond those provided automatically via Facebook are gathered. Various game mechanics are used in addition to social factors to encourage participation, including the awarding of points and badges.

### Participants

**Figure 2:**
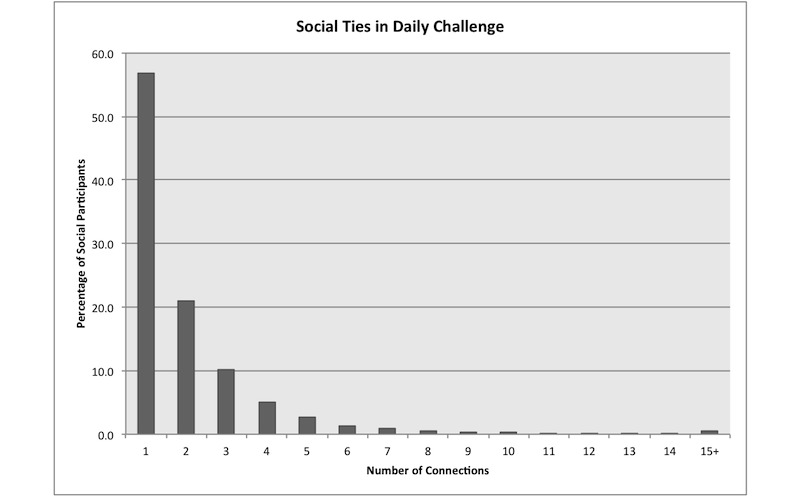
Distribution of the number of connections social participants formed within Daily Challenge.

Participants were Daily Challenge members who registered between September 14, 2010 and July 20, 2011 and who had been members for at least 30 days (N = 128,233). We examined the first 30 days of activity in the program. Individuals whose only interaction with the system was registration were excluded (inactive: n = 43,405 or 33.8%). Members with at least minimal interaction with the system—defined as at least one known email open or site visit after their first emailed challenge (during the 30-day window)—were included in the analysis (active: n = 84,828 or 66.2%). The final dataset comprised 84,828 members who had formed a total of 67,648 personal connections within Daily Challenge. Table 1 summarizes the attributes of the participants in the study. Figure 2 shows the distribution of ties in the program for social users.

The data analyzed in this study are stored in real time in a relational database used for quality improvement purposes. Data include available demographics, self-reported challenge completions, and observed metrics of utilization. All data were anonymized prior to extraction and analysis.

**Table 1:** Attributes of the 84,828 participants in the study.

Attribute		n (%) or mean (SD)	
Days in program, mean (SD)		119.1	(61.6)
Personal connections, mean (SD)		0.8	(1.8)
**Gender, n (%)**			
	Women	71,031	83.7%
	Men	8,712	10.3%
	Unknown	5,085	6.0%

### Process Measures

We obtained engagement measures at the individual level for the following three actions: (1) an email open, (2) a visit to the website, and (3) the completion of a challenge. Participants can perform each of these actions with or without social ties in Daily Challenge.

#### Email Opens

We marked an email as opened if the participant’s email client downloaded the embedded graphical images in the message. This metric undercounts actual reading of the messages, as some email programs either are text based or do not load images. Not every participant subscribed to email delivery. For those who did, they further varied in whether they requested morning emails (a 7 AM message delivering the day’s challenge), reminder emails (sent out at 4 PM if the participant had not yet completed the challenge), or both. Because of the optional and dynamic nature of this feature, we defined email opens as a percentage of days on which a member opened at least one email that was sent to them.

#### Site Visits

Site visit refers to the total number of times a member visited the website. Users are uniquely tracked by identifiers embedded in URLs in email messages, long-term cookies, or, if needed, via a login screen. We recorded a new visit if a page was seen after a period of at least 30 minutes without participant-generated activity while the participant was logged in. Consequently, the maximum number of visits was 47 a day.

#### Challenge Completions

Completions is the number of challenges *reported* by the member as completed. Participants who click the Done link from an email are automatically recorded as having completed the challenge. Alternatively, participants can click the Done button directly on the website to report their challenge completion. Within the context of this study we make no assumptions about the user’s actual behavior in the real world, but rather use the behavior of reporting a completion as a marker of active engagement with the system.

The variables personal connections, email opens, site visits, and completions showed a Pareto distribution, failing to meet the assumption of normality, and were subject to a log-normal transformation prior to analysis. We conducted analyses on transformed individual-level data with the statistical software R [22].

## Results

**Figure 3:**
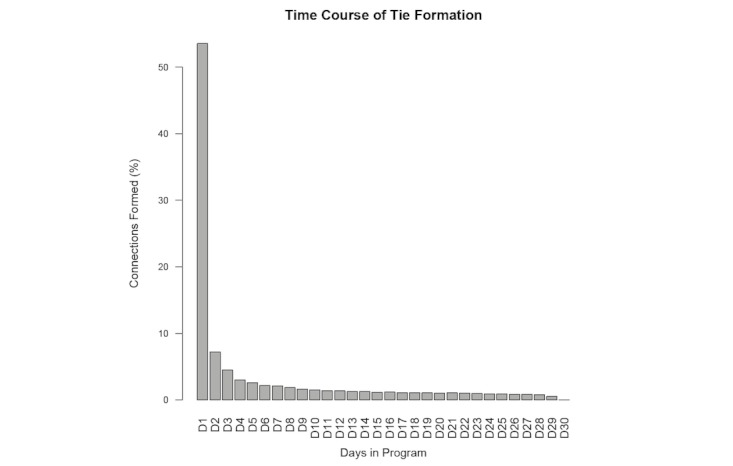
Distribution over time of friendship formation for participants’ first month in Daily Challenge. X-axis: Participants’ days in the program. Y-axis: Percentage of connections formed.

As shown in Figure 3, participants formed the bulk of their ties (n = 50,834 or 75.1%) within 7 days, the overall majority on signup day (n = 36,230 or 53.6%).

We began our investigation of the relation between social ties and engagement by contrasting social and nonsocial experiences of the program. Participants were divided into two groups: participants with at least one personal connection in Daily Challenge (social participants) and those without (nonsocial participants). These groups were compared on each measure of engagement (Table 2 for conditional means).

**Table 2:** Untransformed conditional means for nonsocial and social participants.

	Nonsocial	Social
Email opens (%)	27.2%	33.0%
	(n = 51,775)	(n = 33,021)
Site visits (total)	6.7	12.6
	(n = 51,787)	(n = 33,041)
Completions (total)	6.1	11.0
	(n = 51,787)	(n = 33,041)

Across the board, participants with social ties in the program showed higher engagement. Specifically, participants with personal connections opened more emails sent to them than did participants who had not formed social ties (*t*_65,__198_ = 24.2, *P* < .001, 2-tailed; 95% confidence interval [CI] 5.3−6.2). Furthermore, social participants visited the website more often than their nonsocial counterparts (*t*_51__,__767_ = 61.4, *P* < .001, 2-tailed; 95% CI 5.7−6.0). The pattern was the same with respect to challenge completions, where social participants showed higher rates than nonsocial participants (*t*_57__,__749_ = 75.7, *P* < .001, 2-tailed; 95% CI 4.7−5.0).

**Figure 4:**
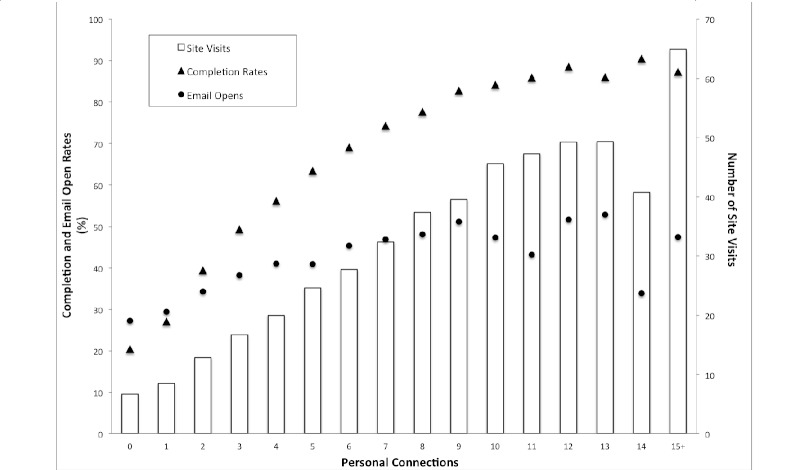
Email opens, site visits, and completion rates per number of personal connections in Daily Challenge. For illustrative clarity, aggregate data are shown. The category 15+ encompasses less than 0.2% of the participants.

We conducted a series of linear regressions to substantiate the relation between social ties and engagement. Figure 4 presents aggregate data on the effect of social ties on each single engagement metric. Predictive models with number of personal connections as the predictor of engagement explained variance in email opens (adjusted *R* *2* = .0094), site visits (adjusted *R* *2* = .1436), and completions (adjusted *R* *2* = .1324).

A positive effect of social ties was found for each measure of engagement. Number of personal connections in the program related to email open rates (beta = 0.68, *P* < .001), site visits (beta = 1.52, *P* < .001), and challenge completions (beta = 1.32, *P* < .001).

## Discussion

In this study, we explored the relation between social ties and engagement in a specific online health behavior intervention. We aimed to determine whether experiencing the intervention socially influenced engagement and whether complex contagion increased engagement. We found compelling evidence that a social experience of the program was associated with improved adherence and program retention, and that engagement level was higher in participants with more social ties in the program.

This social effect affected all measures of engagement examined: email opens, site visits, and reported challenge completions. Participants interacted with the intervention more often if they had social ties, despite the fact that having social ties does not affect a member’s ability to perform the actions studied (or the reward received for each action). The improvement in engagement was substantial: for instance, social participants reported completing an average of 11.0 challenges over 30 days, whereas nonsocial participants reported 6.1 challenge completions for the same period. These findings suggest that social interventions are more engaging than nonsocial ones, and that building social interventions may be part of a solution to the problem of engagement and adherence.

To enable participants to be social, interventions need not require complex, large networks, as a single tie seems to suffice to bolster engagement. However, the social effect was more pronounced for participants with larger social networks, although its benefits seemed to diminish beyond 12 connections, suggesting a natural threshold. More specifically, a higher number of ties was associated with more frequent email opens, site visits, and challenge completions. These results are consistent with the phenomenon of complex contagion, whereby an individual is more likely to adopt a behavior observed in multiple other individuals. By building exposure to others’ behavior into the intervention, we aim to create an environment that enables complex contagion.

Our findings provide early evidence that interventions exploiting social influence can extend to natural, participant-created networks. Online social interventions can leverage participants’ extant networks while providing an environment in which individuals can effectively extend and strengthen their networks by adding new connections. Thus, larger social networks supply the redundancy to facilitate behavior adoption (complex contagion) and the wealth of resources of an enriched network, resources that can satisfy needs otherwise unmet.

Together, our results support the hypothesis that social relationships can affect engagement in an intervention. We note that it is possible that self-reported challenge completions either did not represent true offline behavior or, moreover, had no lasting impact. However, challenge completions, in addition to email opens and site visits, are objective indicators of engagement and dose delivered: participants demonstrated engagement in the action of clicking the Done button. Thus, our findings objectively substantiate the benefits of integrating a social network system on engagement and dose delivered within a health behavior intervention.

There are limitations to this early exploration. The data used were gathered for quality improvement purposes and lack many useful descriptors of participants, including full demographics or indicators of health status. Given that the majority of ties are formed shortly after registration, we chose to treat individual’s social networks as static and the data as cross-sectional; it is possible that the evolution of an individual’s network over time has a different effect on behavior. It also remains unknown to what degree ties can be augmented or encouraged within such a system—in other words, whether individuals’ propensity to form beneficial connections is fixed or mutable.

Most important, it could be the case that individuals more inclined to be adherent initially form social ties, and that these ties are a marker, and not a driver, of adherence and compliance. However, the influence of social ties on one’s attitude and behavior is well documented in social psychology (eg, [14,23,24]) and social network science (eg, [17,25]). The resistance to adopting a risky, costly, or controversial behavior can be overcome by positive reinforcement from multiple independent sources (complex contagion; [17]). Within this model, the continuous exposure of participants to social messages defining active participation as normative leads to an increase in participation proportional to the number of contacts. Our findings are consistent with both this model and highly controlled translational trials in online settings [18] that demonstrate similar findings.

These exciting but initial findings raise several questions on the relation between social ties and engagement. First, without a randomized design, it is difficult to conclusively demonstrate directionality. Importantly, the exact mechanisms of the social effect remain to be determined. For instance, it is unknown whether accountability to one’s ties, social proof of a tie’s activity, or social support (among others) influences engagement. Careful examination of the nature and frequency of social interactions between ties may help tease apart the relative contributions of these potential mechanisms.

The moderators of the social effect should also be examined. The influence of a social tie may be affected by the attributes of the connected individuals or of the connection itself. In particular, social influence may be strongest between participants with shared attributes, or who interact in a specific manner. Moderators may also affect social influence above the individual level, at the community or larger network levels. Furthermore, the topology of an individual’s network, or their position in the network, may modulate social influence. Modern social network methods provide tools to explore these factors and their impact.

Ultimately, these data add weight to the emerging evidence that health behavior change systems structured to include social features can deliver a greater dose of the intervention over time. The careful inclusion of these features, coupled with early and ongoing evaluation, has the potential to augment engagement, retention, and adherence to an intervention, and ultimately drive behavior change.

## Abbreviations

CI: confidence interval

## References

[1] Wantland DJ, Portillo CJ, Holzemer WL, Slaughter R, McGhee EM (2004). The effectiveness of Web-based vs. non-Web-based interventions: a meta-analysis of behavioral change outcomes. J Med Internet Res.

[2] Eysenbach G (2005). The law of attrition. J Med Internet Res.

[3] Christensen H, Griffiths KM, Farrer L (2009). Adherence in internet interventions for anxiety and depression. J Med Internet Res.

[4] Wanner M, Martin-Diener E, Bauer G, Braun-Fahrländer C, Martin BW (2010). Comparison of trial participants and open access users of a web-based physical activity intervention regarding adherence, attrition, and repeated participation. J Med Internet Res.

[5] Leslie E, Marshall AL, Owen N, Bauman A (2005). Engagement and retention of participants in a physical activity website. Prev Med.

[6] Glasgow RE, Nelson CC, Kearney KA, Reid R, Ritzwoller DP, Strecher VJ, Couper MP, Green B, Wildenhaus K (2007). Reach, engagement, and retention in an Internet-based weight loss program in a multi-site randomized controlled trial. J Med Internet Res.

[7] Couper MP, Alexander GL, Zhang N, Little RJ, Maddy N, Nowak MA, McClure JB, Calvi JJ, Rolnick SJ, Stopponi MA, Cole Johnson C (2010). Engagement and retention: measuring breadth and depth of participant use of an online intervention. J Med Internet Res.

[8] Cobb NK, Graham AL, Bock BC, Papandonatos G, Abrams DB (2005). Initial evaluation of a real-world Internet smoking cessation system. Nicotine Tob Res.

[9] Heesch KC, Mâsse LC, Dunn AL, Frankowski RF, Mullen PD (2003). Does adherence to a lifestyle physical activity intervention predict changes in physical activity?. J Behav Med.

[10] Jacobs N, Clays E, De Bacquer D, De Backer G, Dendale P, Thijs H, de Bourdeaudhuij I, Claes N (2011). Effect of a tailored behavior change program on a composite lifestyle change score: a randomized controlled trial. Health Educ Res.

[11] Strecher VJ, McClure J, Alexander G, Chakraborty B, Nair V, Konkel J, Greene S, Couper M, Carlier C, Wiese C, Little R, Pomerleau C, Pomerleau O (2008). The role of engagement in a tailored web-based smoking cessation program: randomized controlled trial. J Med Internet Res.

[12] Rogers EM (1995). Diffusion of Innovations.

[13] Christakis NA, Fowler JH (2007). The spread of obesity in a large social network over 32 years. N Engl J Med.

[14] Cialdini R, Trost M (1998). Social influence: social norms, conformity, and compliance. Gilbert DT, Fiske ST, Lindzey G, editors. The Handbook of Social Psychology. 4th edition.

[15] Cobb NK, Graham AL, Abrams DB (2010). Social network structure of a large online community for smoking cessation. Am J Public Health.

[16] Christakis NA (2008). Health care in a web. BMJ.

[17] Centola D, Macy M (2007). Complex contagion and the weakness of long ties. Am J Sociol.

[18] Centola D (2010). The spread of behavior in an online social network experiment. Science.

[19] (2010). MeYou Health LLC.

[20] Hill JO, Wyatt HR, Reed GW, Peters JC (2003). Obesity and the environment: where do we go from here?. Science.

[21] Hill JO (2009). Can a small-changes approach help address the obesity epidemic? A report of the Joint Task Force of the American Society for Nutrition, Institute of Food Technologists, and International Food Information Council. Am J Clin Nutr.

[22] R Development Core Team (2008). R: A Language and Environment for Statistical Computing: Version 2.13.1 (2011).

[23] Kelman HC (1958). Compliance, identification, and internalization three processes of attitude change. J Conflict Resolut.

[24] Latane B (1981). The psychology of social impact. Am Psychol.

[25] Valente TW (1996). Social network thresholds in the diffusion of innovations. Soc Networks.

